# Effect of implementation of the WHO intrapartum care model on maternal and neonatal outcomes: a randomized control trial

**DOI:** 10.1186/s12884-024-06449-4

**Published:** 2024-04-17

**Authors:** Somayeh Abdolalipour, Shamsi Abbasalizadeh, Sakineh Mohammad-Alizadeh-Charandabi, Fatemeh Abbasalizadeh, Shayesteh Jahanfar, Fatemeh Raphi, Mojgan Mirghafourvand

**Affiliations:** 1 Department of Midwifery, Faculty of Nursing and Midwifery, Tabriz University of Medical Sciences, Tabriz, IR Iran; 2 Women’s Reproductive Health Research Center, Tabriz University of Medical Sciences, Tabriz, Iran; 3 Social Determinants of Health Research Center, Tabriz University of Medical Sciences, Tabriz, IR Iran; 4 Tufts School of Medicine, Department of Public Health and Community Medicine, Boston, USA; 5 Master of Midwifery, Clinical Research Development Unit, Taleghani Hospital, Tabriz University of Medical Sciences, Tabriz, IR Iran; 6 Social Determinants of Health Research Center, Department of Midwifery, Faculty of Nursing and Midwifery, Tabriz University of Medical Sciences, Tabriz, Iran

**Keywords:** Intrapartum care, Childbirth experience, Fear of childbirth, Quality of care

## Abstract

**Background:**

In 2018, the World Health Organization published a set of recommendations for further emphasis on the quality of intrapartum care to improve the childbirth experience. This study aimed to determine the effects of the WHO intrapartum care model on the childbirth experience, fear of childbirth, the quality of intrapartum care (primary outcomes), as well as post-traumatic stress disorder symptoms, postpartum depression, the duration of childbirth stages, the frequency of vaginal childbirth, Apgar score less than 7, desire for subsequent childbearing, and exclusive breastfeeding in the 4 to 6 weeks postpartum period (secondary outcomes).

**Methods:**

This study was a randomized controlled trial involving 108 pregnant women admitted to the maternity units of Al-Zahra and Taleghani hospitals in Tabriz-Iran. Participants were allocated to either the intervention group, which received care according to the ' ‘intrapartum care model, or the control group, which received the’ ‘hospital’s routine care, using the blocked randomization method. A Partograph chart was drawn for each participant during pregnancy. A delivery fear scale was completed by all participants both before the beginning of the active phase (pre-intervention) and during 7 to 8 cm dilation (post-intervention). Participants in both groups were followed up for 4 to 6 weeks after childbirth and were asked to complete questionnaires on childbirth experience, postpartum depression, and post-traumatic stress disorder symptoms, as well as the pregnancy and childbirth questionnaire and checklists on the desire to have children again and exclusive breastfeeding. The data were analyzed using independent T and Mann-Whitney U tests and analysis of covariance ANCOVA with adjustments for the parity variable and the baseline scores or childbirth fear.

**Results:**

The average score for the childbirth experience total was notably higher in the intervention group (Adjusted Mean Difference (AMD) (95% Confidence Interval (CI)): 7.0 (0.6 to 0.8), *p* < 0.001). Similarly, the intrapartum care quality score exhibited a significant increase in the intervention group (AMD (95% CI): 7.0 (4.0 to 10), *p* < 0.001). Furthermore, the post-intervention fear of childbirth score demonstrated a substantial decrease in the intervention group (AMD (95% CI): -16.0 (-22.0 to -10.0), *p* < 0.001). No statistically significant differences were observed between the two groups in terms of mean scores for depression, PTSD symptoms, duration of childbirth stages, frequency of vaginal childbirth, Apgar score less than 7, and exclusive breastfeeding in the 4 to 6 weeks postpartum (*p* > 0.05).

**Conclusion:**

The intrapartum care model endorsed by the World Health Organization (WHO) has demonstrated effectiveness in enhancing childbirth experiences and increasing maternal satisfaction with the quality of obstetric care. Additionally, it contributes to the reduction of fear associated with labor and childbirth. Future research endeavors should explore strategies to prioritize and integrate respectful, high-quality care during labor and childbirth alongside clinical measures.

## Background

The majority of the 140 million childbirths happening each year around the globe are among women with no initial risk factor for themselves or the newborn [1, 2]. Still, childbirth itself is crucial for the survival of both the mother and the infant, as any complication could severely increase the risk of morbidity and mortality. In pursuit of the third goal of sustainable development (To ensure healthy lives and promote well-being for everyone at all ages), global programs have increased their focus on the matter to ensure that women and their newborns would not only survive a potential birth complication but also reach their full potential for a healthy life [3].

Despite the years-long discussions and studies, there is still no standardized definition of " normal” childbirth. Recent studies have suggested that the benchmark for normal labor, devised using the results of studies dating back more than 60 years, may not be suitable for making decisions for every woman [4, 5]. In the past two decades, there has been a considerable surge in the usage of diverse interventions to initiate, accelerate, control, or monitor the physiological process of childbirth, aiming to improve the outcome for both mothers and infants [3]. In some regions, women and newborns die due to limited professional care, while in other regions, they suffer from unnecessary, redundant, or even harmful interventions that stem from medicalized childbirth [6]. This increase in the medicalization of childbirth processes weakens the ' ‘woman’s ability to give birth and negatively affects her experience [3]. Most maternal care policies have recognized that all women and their infants must access evidence-based, fair, compassionate, and respectful care during childbirth. However, what women experience during labor and childbirth in many settings – both high and low-income – is not positive and also, there is not universal access to fundamental interventions [7].

In 2018, recommendations of the World Health Organization (WHO) regarding intrapartum care emphasized the quality of the care, intending to provide recommendations regarding intrapartum a favorable experience. The span of these recommendations goes beyond mortality prevention and includes an attitude based on ' ‘women’s rights during childbirth and optimizing health and comfort for mothers and their infants [6, 7]. In this regard, the WHO recognizes intrapartum care as a platform that allows experienced care providers within a well-functioning system to deliver clinical and non-clinical measures that are respectful, individual-specific, mother-centered, and practical, to optimize the mother’s and the infant’s birth outcomes. To achieve this goal, the WHO has suggested an intrapartum care model that puts the mother and her infant at the center [7]. The nine components of this model are as follows: 1) Respectful labor and childbirth care; 2) Receiving emotional support from a companion of choice; 3) Effective communication with staff; 4) Pain relief strategies; 5) Regular labor monitoring, documentation of events, audit, and feedback; 6) Oral fluid and food intake; 7) Mobility in labor and birth position of choice; 8) Pre-established referral plan; 9) Continuity of care [7].

Healthcare specialists responsible for designing local and national health protocols and directly providing care for pregnant women and their newborns are the primary targets for this model; these include midwives, nurses, general practitioners, obstetricians, and mother and child health program managers [3]. As outlined in the intrapartum care recommendations of the WHO, the experience of the care is as crucial as providing clinical care in reaching the desired individual-based results [8]. However, non-clinical measures during childbirth, such as providing emotional support via companionship, effective communication, and respectful care—which may be relatively inexpensive, are not considered priorities in many healthcare centers [9]. Similarly, obstetric options that respect women’s values and promote decision-making in childbirth’s first and second stages are not constantly provided. These non-clinical aspects of care during labor and childbirth are crucial parts of the care experience and should complement any essential clinical intervention to optimize the quality of the care provided to the woman and her family [3].

The recommendations are based on the idea that by using effective measures during labor and childbirth and by avoiding ineffective (and potentially harmful) interventions, healthcare providers can support women in achieving their desired physical, emotional, and mental outcomes for themselves, their babies, and their family [7]. Efforts for improving respectful maternal care could be counted as one of the admirable and supplementary attributes of a healthcare system, and investing in such fields can have a significant direct impact on the quality of care delivered to mothers [10]. On the other hand, mothers who have experienced low-quality care may be hesitant to use healthcare services for future pregnancies and could also discourage potential users by sharing their negative experiences [11].

Medical interventions have greatly influenced childbirth in Iranian healthcare centers [12]. Furthermore, most Iranian mothers (75%) report one or more instances of non-respectful maternal care. More than half of them stated that they did not even have the right to move during childbirth or choose the most comfortable position [13]. Given the growing agreement among general health specialists that midwifery care has an essential role in providing high-quality service for mothers and infants [14, 15], and also since no study could be found that had analyzed all sections of the WHO’s recommended care model, this study was conducted aiming to determine the effects of implementing this model on the pregnancy experience, fear of childbirth, and the quality of intrapartum care during labor and childbirth (primary outcomes), as well as the duration of childbirth stages, the frequency of vaginal childbirth, Apgar scores less than 7, PTSD (post-traumatic stress disorder) symptoms and postpartum depression, desire for a subsequent pregnancy, and exclusive breastfeeding in the 4 to 6 weeks postpartum period (secondary outcomes).

## Methods

### Study design and participants

This study was the randomized controlled trial section of a thesis (a parallel convergent mixed-methods study) conducted from June 11, 2022, until June 27, 2023. The study population was pregnant women admitted to the Taleghani and Al-Zahra maternity centers. Alzahra Hospital is a Medical Education and Research Center and also, is one of the gynecological and obstetric referral centers in the northwestern region of the country. This center has 10 labor and delivery rooms (LDR). Taleghani Hospital is another Medical Education and Research Center in Tabriz and generally, women with low-risk conditions are admitted there.This center has eight LDRs. Each LDR in these centers is equipped with woman and neonate resuscitation facilities, fetal heart rate monitoring, a warmer, a suction device, a bathroom, and a birth ball. Also, pharmacological pain relief methods are performed at the request of women.

Inclusion criteria included being before the start of the active phase of childbirth and having a childbirth rank of first or second childbirth. The exclusion criteria included indications for cesarean section (e.g., non-cephalic presentation, multiple pregnancies, placenta previa, etc.); obstetric conditions such as post-cesarean vaginal childbirth, pre-eclampsia; underlying conditions such as cardiovascular disease and diabetes, etc.; stillbirth or abnormal fetus; intellectual disability or other mental disorders of the mother; and loss of a close relative in the past three months.

### Sample size

The determination of the sample size for this study was conducted utilizing G-power, focusing on the variables “fear of childbirth” and “childbirth experience”. Referring to findings by Shakarami et al. [16] on fear of childbirth, with assumed values of M1 = 69.3, M2 = 55.44 (anticipating a 20% reduction due to intervention between the measurement phases), SD2 = SD1 = 8.5, two-sided α = 0.05, and Power = 90%, a sample size of 11 individuals was calculated. Furthermore, drawing from Ghanbari Homayi et al.‘s [17] outcomes on the childbirth experience variable, assuming M1 = 2.71, M2 = 3.25 (presuming a 20% increase due to intervention), SD2 = SD1 = 0.73, two-sided α = 0.05, and Power = 95%, a sample size of 49 participants was determined. Considering a 10% dropout rate, the overall sample size calculated for each group was 54 participants.

### Sampling and randomization

Sampling began with women admitted to the maternity ward using the convenience method after receiving the code of ethics (Ethics code: IR.TBZMED.REC.1401.093) and registering at the Iranian Registry of Clinical Trials (Code: IRCT20120718010324N69). After the women received a thorough explanation of the goals and methods of the study, those who were eligible and interested signed a consent form before completing the socio-demographic and obstetric characteristics questionnaires. The women were then assigned to the study groups before entering the active phase of labor. The allocation method was block randomization (stratified based on the number of childbirths, including nulliparous and second childbirth), with block sizes of 4 and 6 and an allocation ratio of 1:1. To ensure allocation concealment, the intervention method was placed inside a series of consecutively numbered opaque envelopes. Due to the nature of the intervention, blinding the researcher and the participants was impossible. However, to ensure the blinding of the data collectors, the post-labor questionnaires were completed with the researchers’ aid. The data were analyzed by a researcher blinded to the study groups. Both groups of women were recruited from both hospitals. Women in the intervention group received the WHO intrapartum care model and those in the control group received the routine care of hospitals but in separate LDRs.

### Intervention and follow-up

The researcher (a midwifery Ph.D. student) provided participants in the intervention group with the intrapartum care model recommended by WHO. With the approval of the head of the maternity ward, care was provided during labor, childbirth, and postpartum until the participant was transferred to the postpartum ward. In cases that needed consultation or faced unexpected high risk, obstetricians involved in the research (second and fourth authors) were contacted. The intervention included the nine components of the WHO-recommended intrapartum care:

1. Respectful labor and childbirth: To apply this part of the intervention, sections of the Sheferaw et al. (2016) respectful care questionnaire were used. The questionnaire’s subdomains indicate that care should be friendly, on time, non-discriminative, and non-abusive [18]. In delivering respectful care, the questionnaire encompasses measures to prevent physical harm or maltreatment, foster empathy, address the mother by name, communicate in a language familiar to her, respect her beliefs and values, adhere to scheduled care delivery, and exhibit non-discriminative behavior throughout the caregiving process.
2. Receiving emotional support from a companion of choice: With the approval of the head of the maternity ward and the hospital’s supervisor, women in the intervention group had a companion of their choice present during labor. In cases where the companion could not be present, the researcher performed the decided tasks with the mother’s approval. As outlined in a previous study [19], the suggested responsibilities of a companion encompass supportive actions (remaining nearby, providing comfort, offering massages, displaying kindness, offering encouragement and motivation). Additionally, expected behaviors in response to signs of fatigue, stress, anxiety, crying, screaming, or other indications of the mother feeling overwhelmed are highlighted. Other recommendations include adhering to guidelines (such as wearing standard attire, refraining from food and tobacco use, avoiding contact with instruments, and notifying personnel when leaving the hospital), as well as recognizing the right to request information from the staff. Additional emphasis was put on respecting other women’s privacy. In the present study, all of these points were mentioned to the companion, and the researcher monitored their execution.
3. Effective communication with staff: the WHO considers communication effective if:

- Women and their families are informed of the evidence, risks, and benefits attributed to methods, procedures, and utilizing/not utilizing technologies and strategies during obstetric care; (b) Effective, respectful, and two-sided communication techniques are employed, and women and their families are respectfully listened to; The women and their families are allowed to take part in the decision-making process according to their personal/familial/cultural preferences [3]. The researcher considered All these items while providing care for the intervention group.

4. Pain relief strategies: In this study, the researcher provided non-pharmacological pain relief techniques such as teaching abdominal breathing with correct inhalation and exhalation, thermotherapy, changing the ' ‘mother’s position, and -with her consent- massage [20]. In cases requiring pharmacological pain relief methods, the obstetrician involved in the study approved and monitored the process.
5. Regular labor monitoring, documentation of events, audit, and feedback: documentation of labor and childbirth events was done by the researcher in addition to the staff. Additionally, the researcher regularly and closely monitored the provided obstetric care during labor, childbirth, and the first two hours after birth, and feedback was given when necessary. Monitoring included periodic fetal heart auscultation using Doppler, controlled uterotonics (oxytocin or misoprostol) and umbilical cord traction to prevent postpartum bleeding, delayed clamping of the umbilical cord, regular monitoring of the mother regarding vaginal bleeding, uterine tone, and vital signs [7] as well as documentation of labor accidents, such as using a partograph form.
6. Oral fluid and food intake: The companions received a list of recommended easily digestible foods and fluids (drinking water, juices, dates, biscuits, and cakes) to obtain. However, they were allowed to consume anything they wished in small and frequent meals.
7. Mobility in labor and birth position of choice: According to a previous study [21], the selected positions for the intervention group in the first stage of childbirth included sitting, walking, semi-sitting, all-fours, and recumbent on the side (both sides). Each of these positions, along with its benefits, was instructed to the mother by the researcher. They were asked to start with the position they felt most comfortable in and remain there for ten minutes before resting for ten minutes between positions. Additionally, they were instructed to have all five positions in mind during 4, 7, and 10-cm dilations. The mobility guideline was based on a former study [22] and advised each woman in the intervention group to walk for an average of one hour during labor, divided into smaller but frequent occasions, by her endurance. If the mother was uncomfortable with this protocol, she decided on her mobility and position during labor by herself.
8. Pre-established referral plan: Higher-level referrals were unnecessary since the study took place in specialty hospitals. Still, regular assessments during labor, the entire childbirth process, and early postpartum were performed for potential cases that required consultation with obstetricians to be identified as soon as possible and appropriate decisions to be made without delay. Due to the vital importance of regular monitoring during labor and childbirth, this section of intervention was provided for both groups of intervention and control.
9. Continuity of care: For women in the intervention group of the present study, continuity of care was in the form of providing care during labor, childbirth, as well as postpartum care (first day) by the researcher in the maternity ward, and on the tenth and fortieth days in the clinic of the educational-therapeutic center (in case of attendance) and (if attendance was not possible) by phone.

Women in the control group received routine care from the maternity ward staff and obstetric residents. This standard care primarily includes medical approaches and attempts to accelerate the process through interventions such as oxytocin injection. Despite the clear guidelines on what can be considered respectful care, not all women receive this kind of respectful care in these centers. Furthermore, the emphasis on medical and clinical care often overshadows the importance of supportive practices, such as allowing the mother to choose her position, encouraging walking during labor, facilitating a companion of choice, promoting the use of non-pharmacological pain relief methods, and engaging in joint decision-making. A companion of the ' ‘woman’s choice is not routinely allowed during childbirth. Additionally, continuity of care by a specific care provider is often not possible, as the labor staff, childbirth agent, and postpartum staff are usually different. In these maternity centers, midwives have to work in an overmedicalized environment, which in addition to taking care of several women, severely limits the opportunities for midwifery-led care. In some cases, non-recommended measures -such as fundal pressure to accelerate the second stage of childbirth- are also implemented.

Throughout and following childbirth, obstetric data, including the active phase duration of labor, durations of the second and third stages of childbirth, and Apgar scores, were documented. Additionally, checklists evaluating the success percentage of interventions were duly completed. A childbirth fear scale was completed once before the active phase (to record the baseline fear of childbirth) and once in 7 to 8 cm dilation. The researcher was the childbirth agent for all women in the intervention group. Participants in both groups were followed up for 4 to 6 weeks after childbirth and completed the pregnancy and childbirth questionnaire (PCQ), childbirth experience, Edinburgh’s postpartum depression scale, and PTSD symptoms scale, as well as the desire for subsequent childbearing and exclusive breastfeeding checklists. The interviews were conducted at the women’s preferred locations, mostly at healthcare centers where they had their electronic files. One of the interviews took place at the clinic of the maternity hospital, where the women had come for postpartum visits and check-ups.

### Study outcomes

#### Primary outcomes

Childbirth experience, intrapartum-care quality, and fear of childbirth.

#### Secondary outcomes

Postpartum depression, PTSD symptoms, duration of childbirth active phase, duration of childbirth second stage, duration of childbirth third stage, normal vaginal childbirth, Apgar score less than 7, desire for subsequent childbearing, and exclusive breastfeeding in the 4 to 6 weeks postpartum period.

### Data collection and scales

#### The socio-demographic characteristic questionnaire

This questionnaire included questions about age, age of spouse, marriage status, duration of the marriage, BMI, woman and her spouse’s education and occupation, housing status, and income status of the family.

#### The obstetric characteristic questionnaire

This questionnaire included questions regarding gestational age, number of pregnancies and childbirths, previous abortions, attending classes during pregnancy, whether the pregnancy is wanted, type of possible previous childbirth, history of difficult labor, spouse support during pregnancy, transfer the baby to NICU, skin to skin contact, breastfeeding in the labor room, and intention to have cesarean in the next childbirth.

#### The success rate of the intervention checklist

This checklist was designed to measure how successful each intervention section was for each ' ‘woman’s labor. The ratio was calculated by providing a yes (if the intervention was made) or a no (if the intervention was not made due to the ' ‘mother’s decision or other reasons). In components of the intervention that are more subjective (such as respectful care, effective communication with staff, and emotional support from a companion of choice), the checklist was completed according to the ' ‘mother’s response, and in other sections by the researcher. The implementation rate of effective communication with the maternity ward staff was 78%; respectful care, emotional support from a companion, regular monitoring of labor and documentation of events, and pre-established referral plan were 100% implementation; continuity of care, mobility in labor and positioning had a success rate of 91.5%, 83% and 87% respectively; oral intake of food and fluids was successful on 96% of occasions; and non-pharmacological and pharmacological pain relief strategies were implemented on 88.2% and 11.8% of cases, respectively.

The childbirth experience questionnaire (CEQ 2.0): This questionnaire measures the childbirth experience and includes 25 statements that cover the following areas: own capacity (feeling of control, personal feelings about childbirth and labor pain), professional support (information and midwifery care), perceived safety (feeling of safe and memories of childbirth), and participation (‘ ‘individual’s ability to change position, be mobile and receive pain relief during labor and childbirth). Twenty-two out of the twenty-five statements are multiple-option questions, and the remaining three are completed using Visual Analogue Scales (VAS). The validity and reliability of this tool were confirmed in the population of American women. Sentences with negative meanings (experience of severe pain, fatigue, fear, and unpleasant memories) are given negative scores. An average of high scores in this tool signals a more positive childbirth experience [23].

#### The delivery fear scale (DFS)

This scale was designed by Wijma et al. and used to measure a ' ‘woman’s fear of childbirth during labor. DFS is a valid self-evaluation questionnaire with ten response levels per item, ranging from 1 (strongly disagree) to 10 (strongly agree). Higher scores indicate greater fear during labor [24].

The pregnancy and childbirth questionnaire (PCQ): This questionnaire is employed to assess mothers’ perspectives on the quality of obstetric and intrapartum care following childbirth. Designed by Truijens et al. in 2014, it is comprised of 25 items: 18 items are based on the women’s experiences and perceptions of pregnant women about the quality of obstetric care, which themselves are divided into two subgroups of personal behavior (11 items; Cronbach’s Alpha = 0.87) and educational information (7 items; Alpha Cronbach = 0.90). The remaining seven items are based on the intrapartum care experiences of women who have recently given birth (Alpha Cronbach = 0.88). Questions are formulated in positive and negative statements, rated on a five-point Likert scale, from totally agree (1) to totally disagree (5). PCQ scores were recoded so that higher points were indicative of higher satisfaction levels. The total range for scores is between 25 and 125 [25]. In this study, only the second half of this questionnaire (experiences of women who have recently given birth regarding intrapartum care) was used.

#### The PTSD symptom scale 1 (PSS_I)

This scale has 17 questions, and using the Likert scale grades the severity of signs for each criterion. The subsets of this tool include (A) signs of re-experiencing (4 questions), (B) signs of avoidance (7 questions), and (C) signs of motivational responses (6 questions). The total range of scores is between 0 and 51 and higher scores were indicative of higher stress levels [26].

#### Edinburgh’s postpartum depression scale

This scale was designed by Cox et al. in 1987 and has implications for measuring depression during pregnancy and after childbirth. This tool is comprised of ten questions with four options each, which in some questions are ordered from low to high severity (items 1, 2, and 4) and in some questions from high to low severity (items 3, 5, 6, 7, 8, 9, and 10). In each question, the participant receives a point from 0 to 3 based on the severity of the symptom; hence the total score will range from 0 to 30. The validity of this tool was calculated by determining the concurrent correlation coefficients of ' ‘Edinburgh’s and ' ‘Beck’s depression scales to be 0.78, and its reliability was calculated using Alpha Cronbach and split-half methods to be 0.75 [27].

#### Partogram

A Partogram is a simple and valid diagram and is often considered the best tool for monitoring the childbirth process and the health status of the mother and her infant. Using this form allows healthcare staff to express the details of the childbirth process visually; this includes information about the ' ‘mother’s health, the health status of the infant, recording the process of childbirth, as well as managing it. A Partogram is a primary alarm mechanism that could significantly facilitate the decision-making process regarding the on-time referral of the mother [28]. Information about the duration of childbirth, natural vaginal childbirth, the implication of oxytocin, analgesia, amniotomy or episiotomy, and the degree of perineal tear, as well as the Apgar score, was elicited from this form.

#### The desire for subsequent childbearing checklist

This checklist is a simple yes or no question regarding the desire to have more children.

#### The exclusive breastfeeding checklist

This checklist is a simple yes or no question on whether or not the newborn received exclusive breastfeeding for the first 4 to 6 weeks after birth.

### Validity and reliability of the utilized tools

In this study, the validity of the socio-demographic and obstetric questionnaires was measured through content and face validity. Psychometrics properties of all the tools used in this study, except for PCQ, including childbirth experience [29], fear of childbirth [30], postpartum depression [31], and PTSD symptoms [26] questionnaires, have been tested and confirmed in Iran. The PCQ’s psychometrics have been tested in another study, which was part of this thesis and currently is underreview.

### Data analysis

The collected data in this study were analyzed using SPSS version 24. A dual data-entry method was adopted. The normal distribution of all data, except for PTSD symptoms and duration of childbirth, was confirmed through a Kolmogorov–Smirnov test. Descriptive statistics, comprising frequencies (percentage) and mean (SD), were utilized to portray socio-demographic and obstetric characteristics. For data with abnormal distribution, medians (25th and 75th percentile) were employed. To compare childbirth experience, postpartum depression, and quality of obstetric care between the two study groups, an Analysis of covariance (ANCOVA) test with parity adjustment was applied. Additionally, the post-intervention fear of childbirth variable was assessed between groups using an ANCOVA test with adjustments for parity and pre-intervention fear of childbirth. To compare the PTSD symptoms and duration of childbirth stages variables between the study groups, a Mann-Whitney U test was performed. To compare the frequency of below 7 Apgar scores, vaginal childbirth, desire for subsequent childbearing, and exclusive breastfeeding, a chi-square test was performed. Since all questionnaires and checklists were completed by the researcher and co-researcher, there was no missing data. Analysis was conducted employing the modified intention-to-treat (ITT) strategy.

## Results

**Fig. 1:**
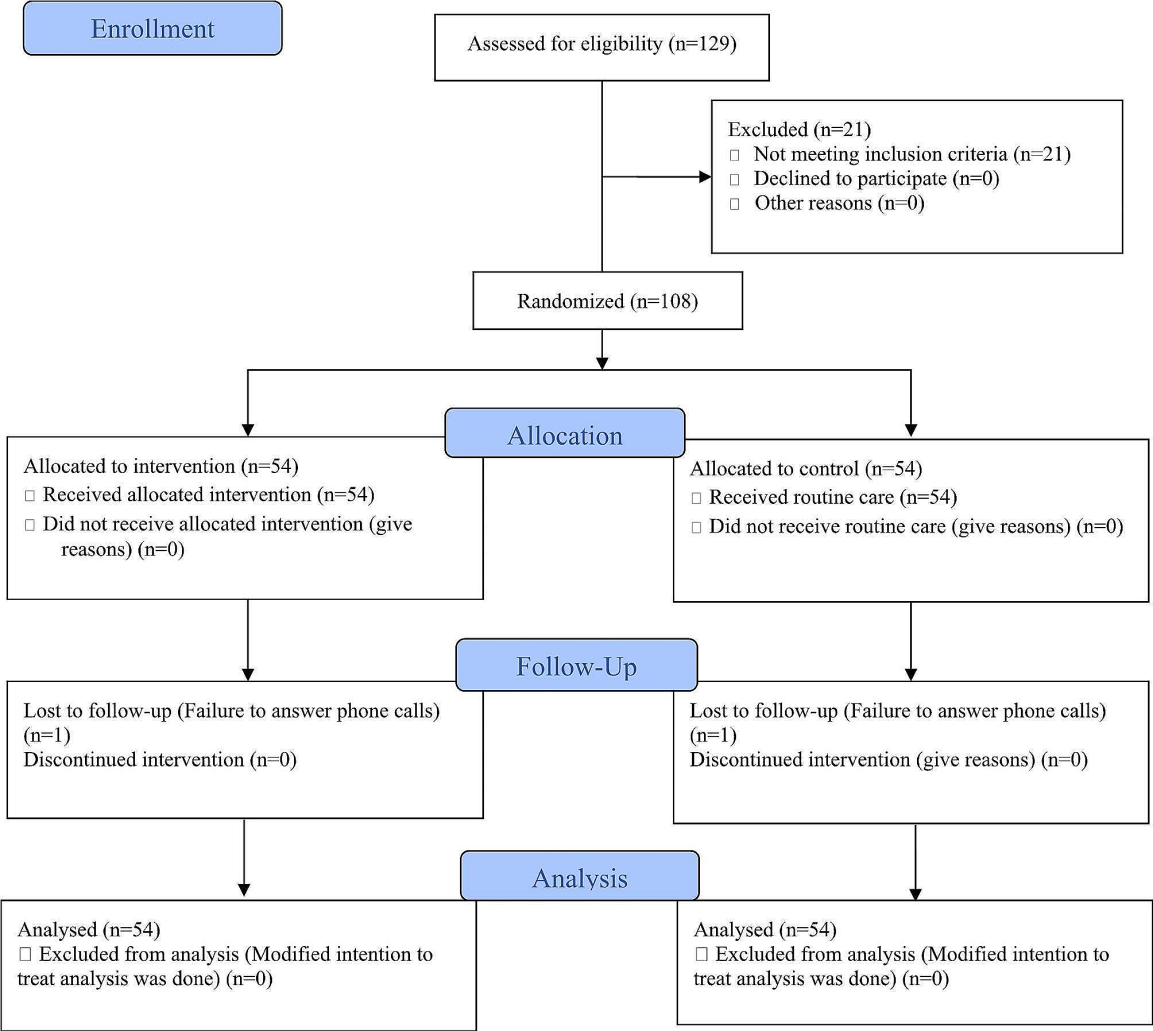
Flow diagram of the study

The sampling process began on June 11, 2022, and was finished on April 5, 2023. The follow-up process ended on May 7, 2023. Initially, 129 mothers who were admitted to the maternity ward were evaluated, 21 of whom were removed from the study due to high-risk pregnancy (high blood pressure, twins) or having their third childbirth or more. There was no case of unwillingness to participate in the study; therefore, the 108 mothers (54 in the control and 54 in the intervention groups) who met eligibility criteria were chosen as samples. Two of the participants (one in either group) were lost to postpartum follow-up, as they did not answer phone calls (Fig. 1).

The mean ± SD, (min–max) of the participants’ ages were 26.2 ± 7, (16–43) and 26.1 ± 6.5 (16–44) in the intervention and control groups, respectively (*p* = 0.966). The percentage of nulliparous was 49.1% in the intervention and 41.8% in the control group (*p* = 0.450) and there was no significant difference in terms of birth ranking. There were no significant differences in terms of other socio-demographic or obstetric characteristics among participants of the two groups (*p* > 0.05). Table 1 shows other socio-demographic attributes of the study groups.

**Table 1:** Socio-demographic and obstetric characteristics of the study participants

Variable	WHO Recommendation	Routine Care	P-value
	(*n* = 54)	(*n* = 54)	
	Mean (SD)	Mean (SD)	
Age (years)	26.2 (7)	26.1 (6.5)	0.966^a^
Spouse age (year)	31.8 (6.1)	32 (5.5)	0.882^a^
Marriage age (year)	5.6 (5.2)	6.6 (5)	0.321^a^
BMI (kg/m^2^)	24.9 (4.2)	25.4 (4.4)	0.534^a^
Gestational age (week)	38.6 (1.4)	38.5 (1.6)	0.533^a^
	**N (%)**	**N (%)**	
**Marital status**			
Married	54 (100)	54 (100)	
**Income**			1.000^b^
Adequate	12 (22.2)	13 (24.1)	
Relatively adequate	37 (68.5)	36 (66.7)	
Inadequate	5 (9.3)	5 (9.3)	
**House status**			0.124^b^
Personal house	22 (41)	34 (63)	
Rented house	18 (33)	10 (18.5)	
Relative house	14 (26)	10 (18.5)	
**Education**			0.209^b^
Primary/Secondary/ High school	30 (55.6)	34 (63)	
Diploma	15 (27.7)	17 (31.4)	
Academic	9 (16.7)	3 (5.6)	
**Spouse education**			0.449^b^
Primary/Secondary/ High school	23 (42.6)	28 (52)	
Diploma	20 (37)	17 (31.5)	
Academic	11 (20.4)	9 (16.7)	
**Job**			0.205^c^
Housewife	49 (90.7)	53 (98.1)	
Employee	5 (9.3)	1 (1.9)	
**Spouse job**			0.842^d^
Employee/Worker	21 (38.9)	19 (35.2)	
Free job	33 (61.1)	35 (64.8)	
**Number of pregnancies**			0.296^b^
1	27 (50)	22 (40.7)	
2	20 (37)	21 (38.9)	
3 and higher	7 (13)	11 (20.4)	
**Number of childbirths**			0.450^d^
First childbirth	26 (49.1)	23 (41.8)	
Second childbirth	27 (50.9)	32 (58.2)	
**Previous abortions**			0.639^d^
Yes	44 (81.5)	41 (75.9)	
No	10 (18.5)	13 (24.1)	
**Participation in childbirth preparation classes**			0.657^d^
Yes	15 (27.8)	12 (22.2)	
No	39 (72.2)	42 (77.8)	
**Unwanted pregnancy**			0.970^d^
Yes	21 (38.9)	13 (24.1)	
No	33 (61.1)	41 (75.9)	
**Type of previous childbirth**			0.137^b^
No childbirth	30 (55.6)	22 (40.7)	
Vaginal	24 (44.4)	31 (57.4)	
Cesarean	0	1 (1.9)	
**History of difficult childbirth**			0.236^d^
Yes	9 (37.5)	7 (21.2)	
No	15 (62.5)	26 (78.8)	
**History of infertility**			0.359^d^
Yes	5 (9.3)	9 (16.7)	
No	49 (90.7)	45 (83.3)	
**Spouse support**			0.518^b^
Low	34 (63)	38 (70.4)	
Moderate	11 (20.3)	9 (16.6)	
Much	9 (16.7)	7 (13)	
**Transfer baby to NICU**			0.805^d^
Yes	9 (79.6)	11 (20.4)	
No	45 (83.3)	43 (16.7)	
**Skin-by-skin contact**			0.436^d*^
Yes	42 (85.7)	39 (78)	
No	7 (14.3)	11 (22)	
**Breastfeeding in the labor room**			0.470^d*^
Yes	40 (81.6)	37 (74)	
No	9 (18.4)	13 (26)	
**Intention to have cesarean in the next childbirth**			< 0.001^d**^
Yes	5 (17.9)	16 (76.2)	
No	23 (82.1)	5 (23.8)	

^a^Independent t-test; ^b^ Chi-square for trend; ^c^Fisher’s exact test; ^d^Chi-square

^*^ Analyses were done for 49 women in the intervention and 50 women in the control group, ^**^Analysis was done for 53 women in each group

The mean ± SD, (min–max) for the childbirth experience total score was 3.5 ± 0.3, (2.8–4) and 2.8 ± 0.4, (1.9–3.5) in the intervention and control groups, respectively, which according to the parity-adjusted ANCOVA test was significantly higher in the intervention group (Adjusted Mean Difference (AMD) (95% CI): 0.7 (0.6 to 0.8), *p* < 0.001). Additionally, all subdomains of childbirth experience were significantly higher in the intervention group compared to the control group (*p* < 0.001). The mean ± SD, (min–max) of the intrapartum care quality score was 31.8 ± 7.1, (28–125) in the intervention group, which is notably higher than the 25.2 ± 7.8, (17–125) of the control group (AMD (95% CI): 7.0 (4.0 to 10), *p* < 0.001). The mean ± SD, (min–max) for the post-intervention fear of childbirth score was 33.5 ± 17.3, (10–76) for the intervention group and 51.7 ± 19.9, (14–86) for the control group, which after adjusting for parity and the baseline fear of childbirth scores, was significantly lower in the intervention group (AMD (95% CI): -16.0 (-22.0 to -10.0), *p* < 0.001). Regarding depression, no significant difference could be reported between the two groups (AMD (95% CI): 0.9 (-1.0 to 3.0), *p* = 0.352) (Table 2).

**Table 2:** Comparison of childbirth experience and its sub-domains, intra-partum care quality, fear of childbirth, and postpartum depression between the study groups

Variable	Intervention (*n* = 53)	Control (*n* = 53)	MD (95%CI)	P-value
	Mean (SD)	Mean (SD)		
CEQ^*^Total (Score range: 1 to 4)	3.5 (0.3)	2.8 (0.4)	0.7 (0.6 to 0.8)	< 0.001^a^
CEQ-Participation	3.7 (0.4)	2.8 (0.6)	0.9 (0.7 to 1.1)	< 0.001^a^
CEQ-Perceived Safety	3.4 (0.4)	2.9 (0.5)	0.6 (0.4 to 0.8)	< 0.001^a^
CEQ-Own Capacity	3(0.5)	2.4 (0.6)	0.6 (0.4 to 0.9)	< 0.001^a^
CEQ-ProfessionalSupport	3.9 (0.2)	3.1 (0.5)	0.8 (0.6 to 0.9)	< 0.001^a^
**Intrapartum care quality** (Score range: 25 to 12)	31.8 (7.1)	25.2 (7.8)	7.0 (4.0 to 10.0)	< 0.001^a^
**Fear of childbirth (pre-intervention)** (Score range: 10 to 100)	39.5 (16.2)	42.8 (17.0)	-2.8 (-9.2 to 3.5)	0.377^b**^
**Fear of childbirth (post-intervention)** (Score range: 10 to 100)	33.5 (17.3)	51.7 (19.9)	-16.0 (-22.0 to -10.0)	< 0.001^c***^
**Postpartum depression** (Score range: 0 to 30)	11.5 (4.9)	10.5 (5.0)	0.9 (-1.0 to 3.0)	0.352^a^

^a^ANCOVA adjusted for parity; ^b^Independent t-test; ^c^ ANCOVA adjusted for parity and pre-intervention fear of childbirth

^*^childbirth experience questionnaire, ^**^Analysis was done for 54 women in each group; ^***^ Analysis was done for 49 women in the intervention and 50 women in the control group

According to the Mann-Whitney U test, there were no significant differences between groups in terms of PTSD symptoms (*p* = 0.166), duration of the active phase (*p* = 0.768), the second stage (*p* = 0.395), or the third stage (*p* = 0.743) of childbirth (Table 3).

**Table 3:** Comparison of duration of labor stages and PTSD^*^ symptoms between the study groups

Variable	Intervention (*n* = 53)		Control (*n* = 53)		P-value^a^
	Mean (SD)	Median (Per 25 to 75)	Mean (SD)	Median (Per 25 to 75)	
The active phase of labor	201.2 (114.1)	180 (120 to 240)	208.4 (117.8)	180 (120 to 240)	0.768^b^
The second stage of labor	28.1 (14.8)	30 (20 to 30)	31.6 (18.0)	30 (15 to 40)	0.395^b^
The third stage of labor	8.4 (6.8)	7.5 (5 to 10)	7.6 (3.2)	5 (5 to 10)	0.743^b^
PTSD^*^symptoms (Score range: 0 to 51)	7.8 (7.5)	8 (2 to 10)	6.4 (6.8)	4 (0 to 9)	0.166

^a^Mann-Whitney U

^b^Analyses were done for 49 women in the intervention and 50 women in the control group

^*^Post-Traumatic Stress Disorder

The number (percentage) of natural childbirth in the intervention and control groups was 49 (90.7%) and 50 (92.6%), respectively, and the difference was not significant (*p* = 0.838). Out of the 54 participants in the intervention group, there were 49 vaginal childbirths and five cesareans, and the control group had 50 vaginal childbirths and four cesareans. The indications for cesarean delivery included one case of bradycardia and, four cases of prolonged first stage in the intervention group, three cases of bradycardia, and one case of meconium-stained amniotic fluid in the control group. There were no infants with a below 7 Apgar score in the intervention group, but three were born in the control group. Still, this difference was not significant between groups (*p* = 0.243). Similarly, the two study groups were not significantly different in terms of desire for subsequent childbearing (*p* = 0.115) and exclusive breastfeeding in the 4 to 6 weeks postpartum period (*p* = 0.473) (Table 4).

**Table 4:** Comparison of type of childbirth, Apgar less than 7, intention to further childbearing and exclusive breastfeeding 4–6 weeks postpartum

Variable	WHO Recommendation	Routine Care	P-value^a^
	(*n* = 53)	(*n* = 53)	
	N (%)	N (%)	
**Type of childbirth**			0.838^*^
Vaginal	49 (90.7)	50 (92.6)	
Cesarean section	5 (9.3)	4 (7.4)	
**Apgar score less than 7**			0.243^*^
Yes	0	3 (5.6)	
No	54 (100)	51 (94.4)	
**Intention to further childbearing**			0.115
Yes	27 (50.9)	18 (34)	
No	26 (49.1)	35 (66)	
**Breastfeeding in 4 to 6 weeks postpartum**			0.473
Yes	44 (83)	40 (75.5)	
No	9 (17)	13 (24.5)	

^a^Chi-square

^*^Analysis was done for 54 women in each group

## Discussion

The present study aimed to assess the effects of the implementation of the WHO-recommended intrapartum care model on some maternal and neonatal outcomes. The results suggested that this intervention could positively affect the childbirth experience, reduce fear of childbirth, and increase ‘mothers’ satisfaction with the quality of intrapartum care, but had no significant statistical effect on depression, PTSD symptoms, duration of childbirth stages, type of childbirth, Apgar score, desire for subsequent childbearing, or exclusive breastfeeding.

In our study, the mean of the total score for the childbirth experience, along with all of its subdomains, was significantly higher in the intervention group. In a study by Demirci et al., ' ‘women’s participation in the provided care during labor and receiving support from a companion and the staff was correlated with a positive childbirth experience [32]. Women’s perception of pain during childbirth and the control they had over themselves during labor and childbirth are among other variables reported to be directly correlated with the childbirth experience [33]. The perceived professional support subdomain received a significantly higher mean score in the intervention group compared to the control group. Previous studies show that support from a midwife is one of the notable factors in this regard, where the actual care is far better than ' ‘women’s expectations. Such support severely depends on the midwife’s and the woman’s relationship and is shown to be the most important factor of intrapartum care [34, 35]. Women in the intervention group scored higher in the participation subdomain as well. Previous studies show that a feeling of personal influence on the surrounding environment and participation in the childbirth process is crucial for a woman to have a positive childbirth experience [34, 36]. Perceived safety was another subdomain where women in the intervention group scored higher than those in the control group. Situations that the mother considers safe, familiar, and supportive will boost oxytocin secretion and the parasympathetic nervous system. This facilitates both the childbirth process and the positive oxytocin-related central actions, consequently boosting positive experiences and emotions [37]. Women in the intervention group scored higher than those in the control group for the personal capacity subdomain. According to a review study by Taheri et al., trials that aimed to prevent unnecessary obstetric interventions through continuous care from a specific midwife and empowerment of women by providing them with the necessary information regarding childbirth saw success in spreading positive maternity experience. Continuing care by a specific midwife improves the ' ‘woman’s childbirth experience through different factors, for example, by providing self-management of pain, the ability to face the challenge of childbirth, control over the process, as well as limiting stressful examinations and interventions [38].

In this study, the quality of intrapartum care as precepted by women was significantly higher in the intervention group than in the control group. Similarly, a study by Fumagalli et al. showed a correlation between interventions during childbirth and a reduction in the care quality as perceived by the mother. Oxytocin induction, epidural analgesia, and instrumental childbirth were associated with low satisfaction, while multiparity correlated to higher intrapartum care quality satisfaction [39]. Conversely, higher mobility during labor and exclusive care by the provider increased ‘mothers’ satisfaction by improving maternal outcomes [40]. Previous studies also show the role of the maternity center and the type of care provided in this regard. Sophisticated hospital equipment, qualified personnel, and a clean hospital environment are commonly reported factors affecting mothers’ satisfaction [41, 42]. However, improvements to the infrastructure and mothers’ health service coverage do not guarantee high-quality service on their own; instead, to encourage childbirth in healthcare facilities and improve the ' ‘mother’s health outcome, the beneficiaries should modify the healthcare system to be more humane, respectful, fair, and responsive to mother’s concerns [11].

After the intervention in the present study, the fear of childbirth score showed a more significant reduction in the intervention group than in the control group. The study by Isbir et al. shows that continuous supporting care during childbirth can effectively reduce the fear of childbirth during the active and transitional phases of delivery. Two factors can explain this effect: first, constant accompaniment eliminates the sense of loneliness and fear of childbirth caused by inadequate support from healthcare specialists. Second, participants who had a higher level of precepted support during childbirth and employed methods that boosted relaxation during the active and transitional phases of childbirth had a more positive attitude toward the supportive intrapartum care they received, which could have consequently reduced their fear of childbirth [43]. Furthermore, mother-oriented childbirth environments that provide a sense of freedom and safety can effectively reduce fear [44]. In Stoll et al.‘s study, women intending to undergo a cesarean section reported fear of childbirth stemming from concerns about pain, the potential impact on their sexual attractiveness, and potential harm to themselves or the baby. Conversely, those intending to have natural childbirth expressed fears related to medical interventions during the process. Furthermore, women who received remarkably satisfactory care from midwives had a noticeably lower fear of childbirth score than those who received care from obstetricians [45]. These findings, along with the results of the present study, show that care providers have a critical role in moderating the fear of childbirth.

In this study, the study groups showed no difference in terms of postpartum depression and PTSD symptoms scores. Generally, psychiatric and social interventions considerably reduce the number of women suffering from postpartum depression. Promising interventions include regular and professional at-home examinations, phone support, and interpersonal psychotherapy [46]. Additionally, in a 2018 study by Capik et al., satisfaction with the healthcare staff’s attitude during childbirth, receiving support, and a positive childbirth experience were negative predictors, and experience of postpartum difficulties by the mother were positive predictors of post-traumatic stress [47]. Still, this disorder is more common among women with previous psychiatric disorders [48]. Anyhow, it could be said that aside from the childbirth experience, numerous other factors can cause PTSD and depression symptoms. A history of depression is one of the important risk factors for postpartum depression in women [49]. Other recognized risk factors include a high-stress lifestyle, lack of social support, domestic violence, and marital dissatisfaction. Cultural characteristics of the family also affect the mother’s mental health status after childbirth. Various cultures have different family structures, and the value attributed to women during pregnancy and after childbirth differs from one culture to another [50]. Additionally, studies have shown that postpartum depression is one of the important predictors of post-traumatic stress, and results in this regard suggest that crucial factors other than childbirth experience and the attitude of staff affect stress [51, 52]. Due to the correlation between a history of psychiatric disorders in women and mental health disorders such as postpartum depression andPTSD, future studies should analyze the effects of such variables by assessing the ' ‘mother’s baseline mental health status.

Not only is there clinical importance to shortening the duration of childbirth, but it is also shown to increase satisfaction and allow mothers and their infants to receive fewer interventions during childbirth, which itself positively affects the outcomes of childbirth [43]. In our study, the duration of childbirth stages had no significant difference between the two groups. However, the results of Dwiarini et al.‘s study showed that perinatal instructions, walking during the first stage, the childbirth status, the infant’s weight, fear of childbirth, and childbirth self-efficacy were the factors affecting the duration of the active phase and second stage of childbirth. Higher levels of self-efficacy and less fear were predictors of the shorter active phase and second stages of childbirth [53]. Moreover, back massage was effective in reducing the duration of the first stage [54], breathing techniques and relaxation shortened the first and second stages [55], and the upright position effectively reduced the duration of the second stage of labor [56]. Perhaps the lack of a significant difference in the duration of labor stages in this study could be attributed to ' ‘oxytocin’s use -to strengthen contractions and accelerate labor- in 78% of cases in the control group, as opposed to 44% in the intervention group.

In the present study, there was no significant difference regarding vaginal and cesarean childbirth frequency between the study groups. Few studies have succeeded in reducing cesarean rates [57]. A study conducted in 32 hospitals in the Canadian city of Quebec managed to yield lower cesarean rates by making interventions such as auditing the indications for cesarean and providing feedback and advice for healthcare specialists; however, the effect size was small (adjusted absolute risk difference = 1.8%) [58]. A study aiming to analyze the correlation between modified intrapartum care and cesarean rates, which was conducted in 39 hospitals in Connecticut and Massachusetts, showed that the presence of a trained midwife alongside the mother at all times, as well as employing less interventionist management approaches (such as limiting the use of IV lines and allowing food consumption during childbirth) were significantly associated with lower cesarean rates [57]. Our ‘study’s lack of a significant difference regarding vaginal delivery rates is probably related to the inadequate sample size for analyzing this objective.

In this study, no statistically significant difference was observed in terms of the inclination for future childbearing between the groups. In a study by Zeng et al., a lower desire level for subsequent pregnancies had a significant connection with a greater fear of childbirth [59]. High levels of fear of childbirth are associated with negative childbirth experiences, and fear of future pregnancies, which probably result in lower desire levels for childbearing in women [60]. Since, in this study, the desire for subsequent childbearing was significantly stronger in nulliparous women compared to multiparous women, the lack of significance in this variable between the two groups of this study could be attributed to multiparous women already having enough children and lack the will to have more.

In this study, there was no significant difference between groups regarding exclusive breastfeeding between 4 and 6 weeks after birth. Starting breastfeeding during the first hour after birth is reportedly one of the factors for successful breastfeeding in the long term [61]. However, primiparity, emotional distress during pregnancy, and cesarean delivery are recognized as independent factors for exclusive breastfeeding for less than two months [62]. Perhaps the lack of a significant difference between the two groups could be explained by the fact that skin-to-skin contact between the mother and her newborn, breastfeeding in the first hours after birth, and breastfeeding instructions and consultations are part of the routine care in both of the centers where this study took place and are provided to all mothers and infants with no clinical complication.

### Strengths and limitations

This is the first study to analyze the effects of all parts of the WHO’s intrapartum care model on maternal and neonatal outcomes. The study’s settings were two of the largest hospitals in Tabriz, with healthcare consumers with the most diverse socioeconomic profiles. This could cause the results to be highly generalizable. Additionally, using the same data collection method (interview) for both groups and low dropout rates in the follow-up phase (1.8%) are other strengths of this study. One of the limitations of this study was that blinding the participants and the researcher was not possible due to the nature of the intervention. However, data collection after childbirth was done by a co-researcher unaware of group allocations. Furthermore, in a handful of cases, care in the intervention group was affected by the routine care provided by hospital staff; for example, the specialist’s decision to begin induction due to prolonged childbirth or the decision to perform an amniotomy. The tool used to analyze care quality was another one of the study’s limitations, as it only analyzed the provided care and not the equipment and facilities of the center. Additionally, data was only collected from women who gave birth in these two centers without any complications, and therefore the results are not generalizable to women with complicated childbirth. The Apgar scores of neonates in the intervention and control groups were not assessed by the same person, which could be regarded as a limitation for the correct analysis of this variable. Therefore, whether neonatal complications differ between these groups should be further evaluated. Data regarding childbirth and the postpartum period was gathered after childbirth via phone interviews rather than in-person, which is another limitation of this study. Another potential limitation could be the lack of a direct question regarding intent to breastfeed, as we assumed that nearly all women plan to breastfeed their baby exclusively. Finally, the intent for subsequent childbearing was assessed by a single question. In future studies, the desire for subsequent pregnancies can be assessed by a scale or other, more standard tools before comparing the results.

## Conclusion

Implementation of the WHO intrapartum care model is effective in reducing the fear of childbirth during labor, improving childbirth experiences, and also improving women’s satisfaction with perceived intrapartum care quality. The priority is to promote a responsive healthcare system for mothers that provides humane, respectful, and fair care. Despite the clear guidelines on what can be considered respectful care and effective communication with staff, not all women receive this kind of care. Additionally, providing this kind of care may have a lower priority compared to clinical care. Further studies should be conducted on how to put respectful and high-quality care during labor and childbirth on the list of priorities alongside clinical measures. Furthermore, studies should focus on finding ways to remove non-recommended measures such as routine induction or fundal hand pressure from clinical settings.

## Data Availability

The datasets generated and/or analyzed during the current study are not publicly available due to limitations of ethical approval involving the patient data and anonymity but are available from the corresponding author at reasonable request.

## Abbreviations

WHO: World Health Organization
CEQ 2.0: Childbirth Experience Questionnaire 2.0
DFS: Delivery Fear Scale
PCQ: Pregnancy and Childbirth Questionnaire
PTSD: Post-Traumatic Stress Disorder
EPDS: Edinburgh Postnatal Depression Scale
VAS: Visual Analog Scale
PSS-I: Post-Traumatic Stress Disorder Scale 1
SD: Standard deviation
K-S: Kolmogorov–Smirnov
NICU: Neonatal Intensive Care Unit
ANCOVA: Analysis of Covariance
AMD: Adjusted Mean Difference
